# Development and Verification of an Autophagy-Related lncRNA Signature to Predict Clinical Outcomes and Therapeutic Responses in Ovarian Cancer

**DOI:** 10.3389/fmed.2021.715250

**Published:** 2021-10-04

**Authors:** Yan Li, Juan Wang, Fang Wang, Chengzhen Gao, Yuanyuan Cao, Jianhua Wang

**Affiliations:** ^1^Department of Obstetrics and Gynecology, The Yancheng Clinical College of Xuzhou Medical University, The First People's Hospital of Yancheng, Yancheng, China; ^2^Department of Obstetrics and Gynecology, The First Affiliated Hospital of Soochow University, Suzhou, China; ^3^Department of Gastroenterology, The Yancheng Clinical College of Xuzhou Medical University, The First People's Hospital of Yancheng, Yancheng, China

**Keywords:** ovarian cancer, autophagy, lncRNAs, signature, prognosis, tumor microenvironment

## Abstract

**Objective:** Long noncoding RNAs (lncRNAs) are key regulators during ovarian cancer initiation and progression and are involved in mediating autophagy. In this study, we aimed to develop a prognostic autophagy-related lncRNA signature for ovarian cancer.

**Methods:** Autophagy-related abnormally expressed lncRNAs were screened in ovarian cancer with the criteria values of |correlation coefficient| > 0.4 and *p* < 0.001. Based on them, a prognostic lncRNA signature was established. The Kaplan–Meier overall survival analysis was conducted in high- and low-risk samples in the training, verification, and entire sets, followed by receiver operating characteristics (ROCs) of 7-year survival. Multivariate Cox regression analysis was used for assessing the predictive independency of this signature after adjusting other clinical features. The associations between the risk scores and immune cell infiltration, PD-L1 expression, and sensitivity of chemotherapy drugs were assessed in ovarian cancer.

**Results:** A total of 66 autophagy-related abnormally expressed lncRNAs were identified in ovarian cancer. An autophagy-related lncRNA signature was constructed for ovarian cancer. High-risk scores were indicative of poorer prognosis compared with the low-risk scores in the training, verification, and entire sets. ROCs of 7-year survival confirmed the well-predictive efficacy of this model. Following multivariate Cox regression analysis, this model was an independent prognostic factor. There were distinct differences in infiltrations of immune cells, PD-L1 expression, and sensitivity of chemotherapy drugs between high- and low-risk samples.

**Conclusions:** This study constructed an autophagy-related lncRNA signature that was capable of predicting clinical outcomes and also therapeutic responses for ovarian cancer.

## Introduction

Ovarian cancer represents the major cause of death among gynecological malignancies (1). Surgery followed by chemotherapy (such as platinum and taxane) remained the first-line therapeutic strategy (2). Approximately 80% of the subjects originally respond to this therapy. Nevertheless, the majority of the subjects in late stages usually experienced recurrence following chemotherapy, thereby leading to an undesirable prognosis (5-year survival <50%). Hence, it is significant to probe into the pathogenesis of ovarian cancer and also predictive indicators for prognostic stratification.

Alterations in gene expression profiling have become fundamental laboratory tools for improving tumor diagnoses, survival outcomes, and also treatment responses, which overcome weaknesses of typical clinical and imaging features due to heterogeneity at the genetic and molecular levels (3). Dysregulated long non-coding RNAs (lncRNAs) such as LINC00189, CACNA1G-AS1, and CHRM3-AS2 have been implicated in ovarian cancer initiation and the progress, highlighting their promising functions as markers of precision medicine (4). Their correlations to survival outcomes and treatment responses are still indistinct in ovarian cancer. A few lncRNA-based expression signatures have been developed for predicting survival outcomes, status, and chemosensitivity in ovarian cancer. For instance, Zheng et al. developed a three-lncRNA signature (LOC101927151, LINC00861, and LEMD1-AS1) for predicting clinical outcomes of the subjects with ovarian cancer on the basis of copy number variation (5). Zhang et al. proposed a three-lncRNA signature (LINC01619, DLX6-AS1, and AC004943.2) that can predict survival and response of chemotherapy in ovarian cancer (6). In comparison to abundant lncRNAs identified by genome-wide studies, functionally lncRNAs require better characterization in ovarian cancer.

Autophagy, an evolutionarily conserved process, maintains cell homeostasis through a lysosomal degradation system that supports cell survival and also maintains homeostasis under various types of stress (7). Autophagy-based cell deaths provide molecular mechanisms and clinical implications upon ovarian cancer therapy (8). Thus, it is of significance to identify key regulators of autophagy for theoretical basis and clinical practice. Several studies have found that autophagy can be mediated by lncRNA regulators in ovarian cancer (9–12). For instance, GAS8-AS1 inhibits the progress of ovarian cancer by activation of autophagy through binding with Beclin1 (9). Moreover, lncRNA TUG1 induces autophagy-related paclitaxel resistance *via* sponging miR-29b-3p in ovarian cancer (10). Also, silencing HOTAIR enhances the sensitivity to cisplatin in ovarian cancer via inhibition of cisplatin-induced autophagy (11). LncRNA highly upregulated in liver cancer exerts a carcinogenic effect *via* targeting autophagy-related genes ATG7 and ITGB1 in an epithelial ovarian cancer (12). Thus, autophagy-related lncRNAs possess potential as prognostic indicators and therapeutic targets.

Ovarian cancer represents an insidious malignancy, which usually develops asymptomatically to late stages along with metastases, chemoresistance, and undesirable clinical outcomes (13). Autophagy is a key bioprocess during the initiation and progression of ovarian cancer (14). LncRNA regulators are involved in the autophagy process. There is still a lack of systematic analysis for identifying autophagy-related lncRNA signature for prediction of the survival outcomes of the patients with ovarian cancer. Herein, this study developed a prognostic autophagy-related lncRNA signature for ovarian cancer.

## Materials and Methods

### Data Retrieval and Pre-processing

Among all databases, only the Cancer Genome Atlas (TCGA; https://portal.gdc.cancer.gov/) database has the RNA-seq expression profiling and corresponding clinical and prognostic information of ovarian cancer. Thus, we curated transcriptome data and clinical information containing age, survival time, recurrence, survival status, histologic grade, and pathologic stage of 379 ovarian cancer tissues from the TCGA database. Mutation annotation format (MAF) files of somatic mutation data of ovarian cancer samples were also downloaded from the TCGA database. Meanwhile, the RNA-seq expression profiles of 133 normal ovarian samples were obtained from the genotype tissue expression (GTEx; https://toil.xenahubs.net/download/GTEX_phenotype.gz) database (15). The RNA-seq counts values from the TCGA and GTEx databases were normalized and preprocessed by the TCGAbiolinks package (16). Based on the HUGO Gene Nomenclature Committee (HGNC; http://www.gene.ucl.ac.uk/cgi-bin/nomenclature/searchgenes.pl), lncRNAs and mRNAs were annotated (17).

### Acquisition of Abnormally Expressed lncRNAs

Abnormally expressed lncRNAs between ovarian cancer and normal ovarian specimens were screened utilizing edgeR package (http://bioconductor.org) based on gene expression data (18). Adjusted *p* ≤ 0.05 and |log_2_fold change (FC)| ≥ 1 were set as the criteria values of abnormally expressed lncRNAs.

### Acquisition of Autophagy-Related lncRNAs

A total of 232 autophagy-related genes were retrieved from the Human Autophagy Database (HADb; http://www.autophagy.lu/). The correlation between abnormally expressed lncRNAs and autophagy-related genes was analyzed by psych package (https://CRAN.R-project.org/package=psych) with Pearson's correlation analysis. Autophagy-related lncRNAs were screened with the criteria values of |correlation coefficient| > 0.4 and *p* < 0.001.

### Construction of a LASSO Regression Model

By adopting the least absolute shrinkage and selection operator (LASSO) Cox regression analysis, this study constructed an autophagy-related lncRNA model utilizing the glmnet package (19). The association between the lncRNAs in this model and overall survival (OS) was assessed by univariate Cox regression analysis. LncRNAs with hazard ratio (HR) > 1 and *p* < 0.05 were risk factors, while those with HR <1 and *p* < 0.05 were protective factors. The risk score of each sample was determined. The formula was as follows: risk score = coefficient (lncRNA1) × expression (lncRNA1) + coefficient (lncRNA2) × expression (lncRNA2) + … + coefficient (lncRNA*n*) × expression (lncRNA*n*). The distributions of risk scores, survival status, and disease progress were assessed in the samples of ovarian cancer.

### Assessment of the Prognostic Model

All the subjects with ovarian cancer were randomly separated into the training set and the validation set at a ratio of 1:1. The subjects with ovarian cancer were separated into high- and low-risk groups. The Kaplan–Meier survival analyses were carried out to assess the OS differences between high- and low-risk groups with survival package in the training set, validation set, and the whole dataset. Time-dependent receiver operating characteristic (ROCs) curves were depicted for estimating the predictive efficacy of survival time through risk score and other clinical features (age, pathologic stage, and histologic grade) utilizing the survival ROC package. Furthermore, the differences in the risk scores between patients with different histologic grades or pathologic stages were analyzed by one-way ANOVA. Multivariate Cox regression analysis was presented for evaluating whether the risk score could be independent of other clinical features including pathologic stage and histologic grade.

### CIBERSORT

CIBERSORT algorithm (http://cibersort.stanford.edu/) can characterize cell compositions of complex tissues based on the gene expression profiles (20). This algorithm is superior to other methods in terms of noise, unknown mixture content, and closely related cell types. CIBERSORT algorithm was employed to characterize immune cell compositions (including B cells naïve, B cells memory, plasma cells, T cells CD8, T cells CD4, naïve T cells, CD4 memory resting, T cells CD4 memory activated, T cells follicular helper, T cells regulatory (Tregs), T cells gamma delta, NK cells resting, NK cells activated, monocytes, macrophages M0, macrophages M1, macrophages M2, dendritic cells resting, dendritic cells activated, mast cells resting, mast cells activated, eosinophils and neutrophils) in ovarian cancer tissues.

### Estimation of Stromal and Immune Cells in Malignant Tumor Tissues Using Expression Data

The fractions of stromal and immune cells were inferred in each ovarian cancer sample using the ESTIMATE algorithm (https://sourceforge.net/projects/estimateproject/) (21). The differences in immune and stromal scores were assessed between high- and low-risk samples of ovarian cancer by Student' *t*-test.

### Sensitivity to Chemotherapy Drugs

A total of 94 ovarian cancer-related chemotherapy drugs were collected from the Cancer Genome Project (CGP; version 2014). The pRRophetic package was employed to predict the clinical chemotherapeutic responses based on the gene expression profiles of ovarian cancer (22). The IC50 values of chemotherapy drugs were compared between high- and low-risk groups utilizing Student's *t*-test.

### Somatic Mutation Analysis

The “Masked Somatic Mutation” data were obtained and processed with VarScan software (version 2), a method for the detection of somatic mutation and copy number variation in exome data (23). The maftools package (24) was adopted to analyze the MAF of the somatic variants. The “titv” function classified single nucleotide polymorphisms (SNPs) into transitions (Ti) and transversions (Tv). The overall distribution of the six different SNVs and the proportion of transitions in each sample were evaluated. The oncoplot of the top 30 mutated genes was depicted by the “ComplexHeatmap” function. The “somaticInteractions” function was used to detect mutually exclusive or co-occurring genomes, which were tested by Fisher's exact test.

### Gene Set Enrichment Analysis

Pathways underlying the autophagy-related lncRNA signature were evaluated through GSEA package (25). The “c5.bp.v6.2.symbols.gm” gene set was curated from the Molecular Signatures Database, which was employed as a reference set. Terms with |nominal enrichment score (NES)| > 1.7 and nominal *p* < *0.05* were significantly enriched.

## Results

### Screening Abnormally Expressed lncRNAs

**Figure 1:**
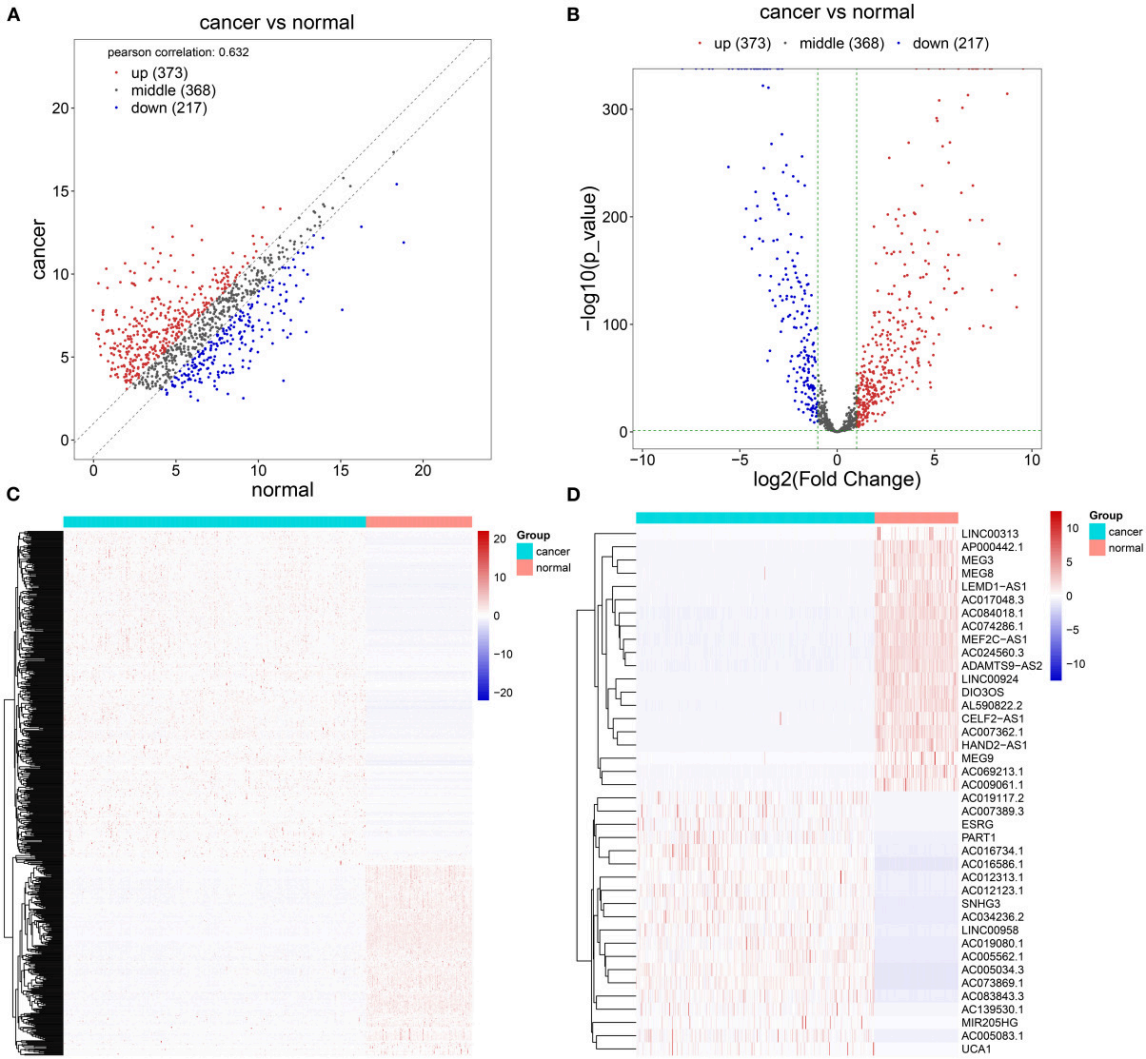
Screening abnormally expressed long noncoding RNAs (lncRNAs) between ovarian cancer and normal ovarian specimens using TCGA and GTEx datasets. **(A–C)** Scatter, volcano, and heatmap diagrams for the up (red) and downregulated (blue) lncRNAs in ovarian cancer and normal samples. **(D)** Heatmap for the top 20 up and downregulated lncRNAs for ovarian cancer.

Herein, we retrieved RNA expression profiles of ovarian cancer and normal ovarian tissues from TCGA and GTEx datasets. Totally, 590 lncRNAs with adjusted *p* ≤ 0.05 and |log_2_FC| ≥ 1 were abnormally expressed in 379 specimens of ovarian cancer in comparison with the normal ovarian cancer (Figures 1A–C). There were 373 upregulated (Supplementary Table 1) and 217 downregulated (Supplementary Table 2) lncRNAs for ovarian cancer. We separately displayed the top 20 upregulated (Table 1) and downregulated (Table 2) lncRNAs for ovarian cancer (Figure 1D).

**Table 1:** The top 20 upregulated lncRNAs in ovarian cancer.

**Gene name**	**log_**2**_FC**	***P*-value**	***Q*-value**	**Cancer**	**Normal**
AC012313.1	9.547183939	0	0	10.31895834	0.771774397
UCA1	9.216655018	1.2272E-116	6.3207E-116	12.81661174	3.59995672
AC007389.3	9.147273622	1.8891E-146	1.321E-145	9.442887756	0.295614134
LINC00958	8.733161562	0	0	10.64514913	1.911987563
AC139530.1	8.320256095	1.2565E-175	1.0943E-174	9.16409823	0.843842135
AC019117.2	7.940574318	1.5611E-132	9.4059E-132	10.17086201	2.230287692
AC073869.1	7.93835612	0	0	11.53335685	3.595000733
ESRG	7.88342569	1.29304E-97	5.43305E-97	9.509050129	1.625624439
AC034236.2	7.840261744	0	0	7.803144412	−0.037117331
AC012123.1	7.575128788	0	0	10.06414233	2.489013539
MIR205HG	7.508354236	2.9168E-99	1.25304E-98	10.64232063	3.133966398
PART1	7.450907876	1.1583E-197	1.1681E-196	12.24750023	4.796592357
AC005562.1	7.220023466	0	0	7.809237126	0.58921366
AC016586.1	7.200189856	0	0	11.25391667	4.053726816
AC016734.1	7.136519504	0	0	7.696281571	0.559762067
AC083843.3	7.119307279	0	0	9.449229653	2.329922374
AC019080.1	6.989829409	0	0	7.558546463	0.568717055
AC005034.3	6.970382156	0	0	9.402961692	2.432579536
AC005083.1	6.968474931	9.7814E-230	1.2628E-228	9.411351502	2.442876571
SNHG3	6.926312525	0	0	12.89759844	5.971285912

**Table 2:** The 20 downregulated lncRNAs in ovarian cancer.

**Gene name**	**log_**2**_FC**	***P*-value**	***Q*-value**	**Cancer**	**Normal**
AC007362.1	−7.953839402	0	0	3.573056805	11.52689621
DIO3OS	−7.25010061	0	0	7.8433415	15.09344211
MEG3	−6.926193554	0	0	11.89962972	18.82582328
LINC00313	−6.572300069	0	0	2.518410795	9.090710864
HAND2-AS1	−6.400004123	0	0	6.508966669	12.90897079
MEG9	−5.584769193	5.2013E-247	7.3277E-246	6.183950367	11.76871956
AC024560.3	−5.562626374	0	0	6.396148069	11.95877444
AC074286.1	−5.375749227	0	0	2.776822389	8.152571616
AL590822.2	−5.097997242	0	0	4.32297655	9.420973791
LINC00924	−5.04563661	0	0	5.818215591	10.8638522
ADAMTS9-AS2	−4.904513345	0	0	6.606317799	11.51083114
MEG8	−4.756706248	5.3224E-182	4.9503E-181	4.749036786	9.505743034
LEMD1-AS1	−4.748134967	0	0	7.224444409	11.97257938
AC017048.3	−4.676146545	3.2389E-208	3.6939E-207	5.295737783	9.971884328
AC069213.1	−4.643176366	0	0	4.407805217	9.050981583
AP000442.1	−4.428047311	0	0	3.056508739	7.48455605
CELF2-AS1	−4.38133152	5.0736E-171	4.3013E-170	4.075613874	8.456945394
MEF2C-AS1	−4.276829555	0	0	4.792378869	9.069208425
AC009061.1	−4.255822376	0	0	4.029896192	8.285718568
AC084018.1	−4.237903564	0	0	8.332160874	12.57006444

### Construction of an Autophagy-Related lncRNA Prognostic Signature in Ovarian Cancer

**Figure 2:**
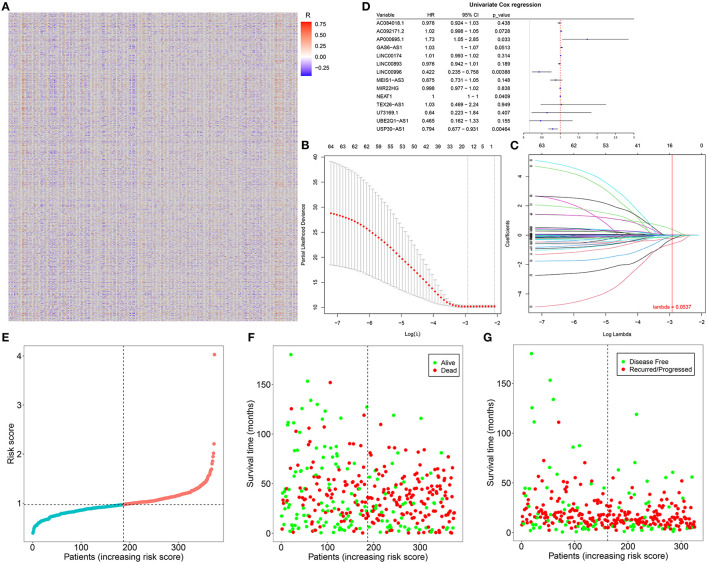
Construction of an autophagy-related lncRNA signature in ovarian cancer. **(A)** Heatmap for the correlation between abnormally expressed lncRNAs and autophagy-related genes in ovarian cancer specimens. Red indicates positive correlation while blue indicates negative correlation. **(B)** LASSO coefficient profiles based on autophagy-related abnormally expressed lncRNAs in ovarian cancer samples. **(C)** Selecting the optimal parameter (λ) in the LASSO model. **(D)** Forest plots for the univariate Cox regression analysis results of autophagy-related abnormally expressed lncRNAs. **(E)** The distributions of the risk scores of patients with ovarian cancer. **(F,G)** The distributions of survival status (green: alive and red: dead) and disease progress (green: disease-free and red: recurred/progressed) for the patients with ovarian cancer. Black dotted line indicates the median value of the risk scores.

The correlation between abnormally expressed lncRNAs and autophagy-related genes was evaluated by Pearson's correlation analysis, which was visualized into the heat map (Figure 2A; Supplementary Table 3). With the criteria values of |correlation coefficient| > 0.4 and *p* < 0.001, we selected 66 autophagy-related abnormally expressed lncRNAs, as shown in Table 3. Using LASSO regression analysis, we determined the regression coefficients of the 14 lncRNAs in the model (Figures 2B,C). The risk score of each specimen of ovarian cancer was calculated as follows: AC084018.1 expression ^*^ (−0.008315282) + AC092171.2 expression ^*^ 2.84E-05 + AP000695.1 ^*^ 0.395692194 + GAS6-AS1 expression ^*^ 0.028077942 + LINC00174 expression ^*^ 0.010226196 + LINC00893 expression ^*^ (−0.013179597) + LINC00996 expression ^*^ (−0.214479586) + MEIS1-AS3 expression ^*^ (−0.093561265) + MIR22HG expression ^*^ (−0.007661823) + NEAT1 expression ^*^ (0.000320475) + TEX26-AS1 expression ^*^ (−0.061717576) + U73169.1 expression ^*^ (−0.263275905) + UBE2Q1-AS1 expression ^*^ (−0.589381398) + USP30-AS1 expression ^*^ (−0.114206463). Univariate Cox regression analysis results showed that AP000695.1 (HR: 1.73, 95% CI: 1.05–2.85, *p*-value: 0.033) was a risk factor of ovarian cancer while LINC00996 (HR: 0.422, 95% CI: 0.235–0.758, *p*-value: 0.00388) and USP30-AS1 (HR: 0.794, 95% CI: 0.677–0.931, *p*-value: 0.00464) were protective factors of ovarian cancer (Figure 2D). In Figure 2E, we visualized the distributions of the risk scores of the patients with ovarian cancer. Also, we found that the risk scores displayed associations with survival status (Figure 2F) and disease progression (Figure 2G).

**Table 3:** A total of 66 autophagy-related abnormally expressed lncRNAs.

**LncRNAs**	**LncRNAs**	**LncRNAs**
AC005387.2	C9orf163	LINC00996
AC006538.1	CACTIN-AS1	MAP3K14-AS1
AC007292.3	CCDC39	MEG3
AC007382.1	CDKN2B-AS1	MEIS1-AS3
AC009093.1	DHRS4-AS1	MIR22HG
AC010336.1	EHMT2-AS1	MKNK1-AS1
AC073869.1	ERVK13-1	NARF-IT1
AC084018.1	EXTL3-AS1	NEAT1
AC092171.2	FAM13A-AS1	PCED1B-AS1
AC105020.1	FLNB-AS1	PRKCQ-AS1
AC137932.1	GAS6-AS1	RNF216P1
ACTA2-AS1	HCP5	SH3BP5-AS1
AL136115.1	LINC00174	SPANXA2-OT1
AL137127.1	LINC00265	SRGAP3-AS2
AL590822.2	LINC00342	TEX26-AS1
AP000695.1	LINC00624	TNK2-AS1
AP005482.1	LINC00677	TTLL10-AS1
AP006621.1	LINC00702	U73169.1
AP4B1-AS1	LINC00893	UBE2Q1-AS1
ARAP1-AS2	LINC00894	USP30-AS1
C10orf55	LINC00954	YEATS2-AS1
C1orf195	LINC00968	ZSWIM8-AS1

### Evaluation of the Autophagy-Related lncRNA Signature as a Robust Prognostic Factor in Ovarian Cancer

**Figure 3:**
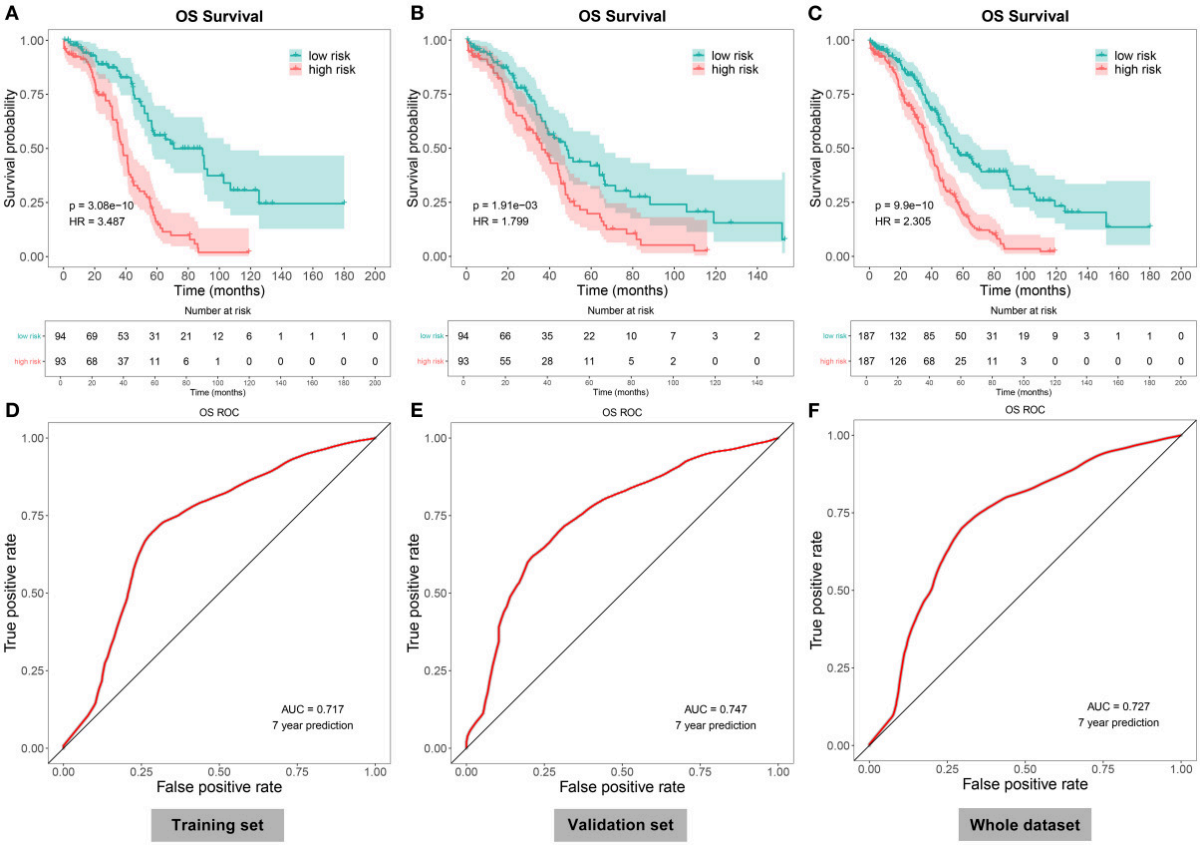
Evaluation of the autophagy-related lncRNA signature as a robust prognostic factor in ovarian cancer. **(A–C)** The Kaplan–Meier overall survival curves between high- and low-risk patients with ovarian cancer in the **(A)** training set, **(B)** validation set, and **(C)** the whole dataset. *P*-values for log-rank tests. **(D–F)** Receiver operating characteristics (ROCs) under 7-year survival in the **(D)** training set, **(E)** validation set, and **(F)** the whole dataset.

Herein, all the patients with ovarian cancer were randomly separated into the training set and validation set (both *n* = 187) in Table 4. Based on the median value of the risk scores, the patients were divided into high- and low-risk groups. Our data showed that there were distinct differences in OS time between the two groups in the training set (*p* = 3.08e-10), validation set (*p* = 1.91e-03), and the whole dataset (*p* = 9.9e-10; Figures 3A–C). Patients in the low-risk group had prolonged OS duration in comparison with those in the high-risk group. ROCs were conducted to validate the predictive performance of this signature. The area under the curves under 7-year survival were 0.717, 0.747, and 0.727 in the training set, validation set, and the whole dataset, respectively (Figures 3D–F).

**Table 4:** The clinical characteristics of patients with ovarian cancer.

**Variables**	**The whole set**	**Training set**	**Validation set**
	**(*N* = 374)**	**(*N* = 187)**	**(*N* = 187)**
Age	59.54 ± 11.4	60.34 ± 10.63	58.74 ± 12.09
**Status**			
Alive	145 (38.77)	73 (39.04)	72 (38.5)
Dead	229 (61.23)	114 (60.96)	115 (61.5)
**Pathologic stage**			
Stage I	1 (0.27)	0 (0)	1 (0.53)
Stage II	22 (5.88)	9 (4.81)	13 (6.95)
Stage III	291 (77.81)	139 (74.33)	152 (81.28)
Stage IV	57 (15.24)	36 (19.25)	21 (11.23)
Unknow	3 (0.8)	3 (1.6)	0 (0)

### The Autophagy-Related lncRNA Signature as an Independent Prognostic Factor for Ovarian Cancer

**Figure 4:**
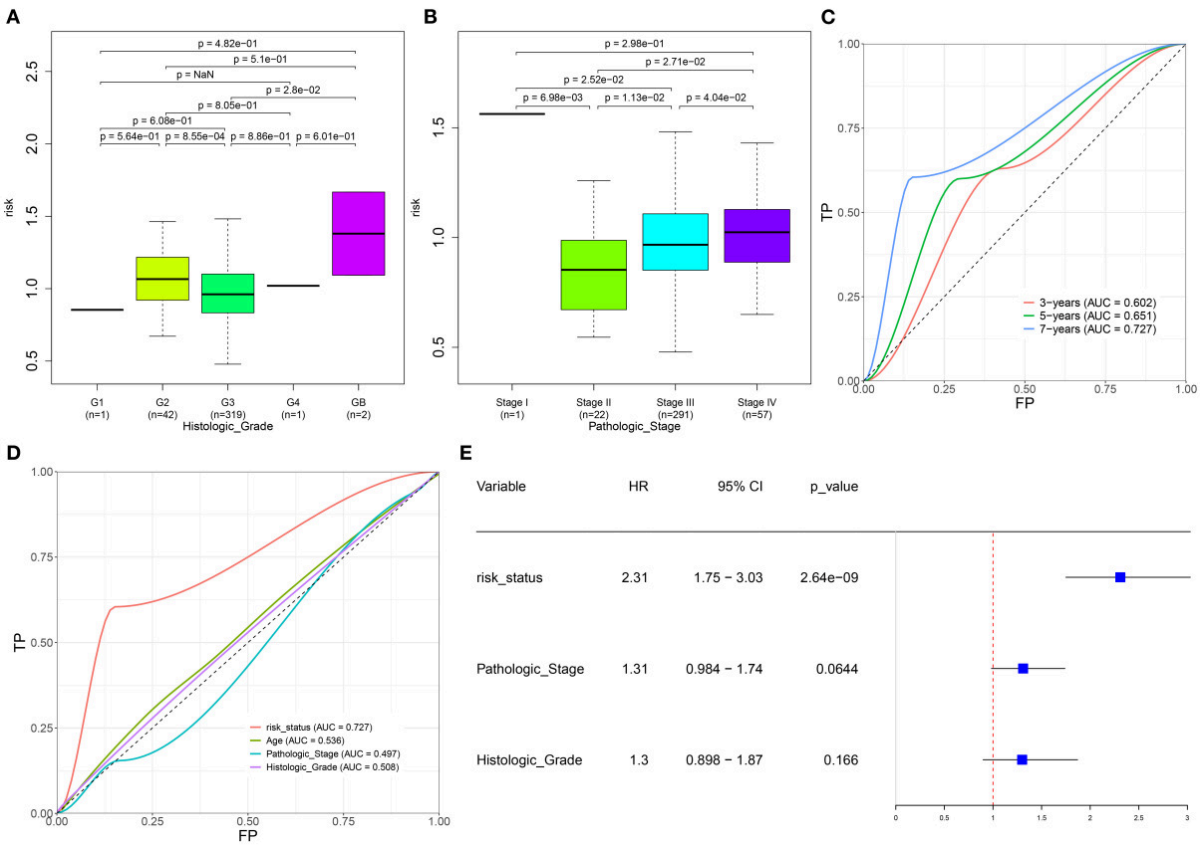
Evaluation of the autophagy-related lncRNA signature as an independent prognostic factor for ovarian cancer. **(A,B)** The distributions of the risk score in patients with ovarian cancer with different **(A)** histologic grades and **(B)** pathologic stages. **(C)** AUCs under 3-, 5-, and 7-year survival for the risk score. **(D)** AUCs under 7-year survival for the risk scores and other clinical features (age, pathologic stage, and histologic grade). **(E)** Multivariate Cox regression analysis of the risk score, pathologic stage, and histologic grade.

We further evaluated the correlations between the risk scores and other clinical features in specimens with ovarian cancer. As a result, lowered risk scores were detected in grade 3 than grade 2 (*p* = 8.55e-04; Figure 4A). Meanwhile, we found that the risk scores increased gradually as the pathologic stages increased (Figure 4B). The AUCs under 3-, 5-, and 7-year survival time were 0.602, 0.651, and 0.727 for the patients with ovarian cancer, indicating the well-predictive performance of this signature (Figure 4C). Furthermore, compared with the other clinical characteristics including age (AUC = 0.536), pathologic stage (AUC = 0.497), and histologic grade (AUC = 0.508), there was a higher AUC value under 7-year OS time for the risk score (Figure 4D). The data suggested that this risk score might possess higher sensitivity and accuracy in predicting the prognosis of the patients with ovarian cancer. Multivariate Cox regression analysis demonstrated that this risk score could be independently predictive of the prognosis of the patients (HR: 2.31, 95% CI: 1.75–3.03, *p* = 2.64e-09; Figure 4E).

### The Autophagy-Related lncRNA Signature Is Associated With Immune Cell Infiltration in Ovarian Cancer

**Figure 5:**
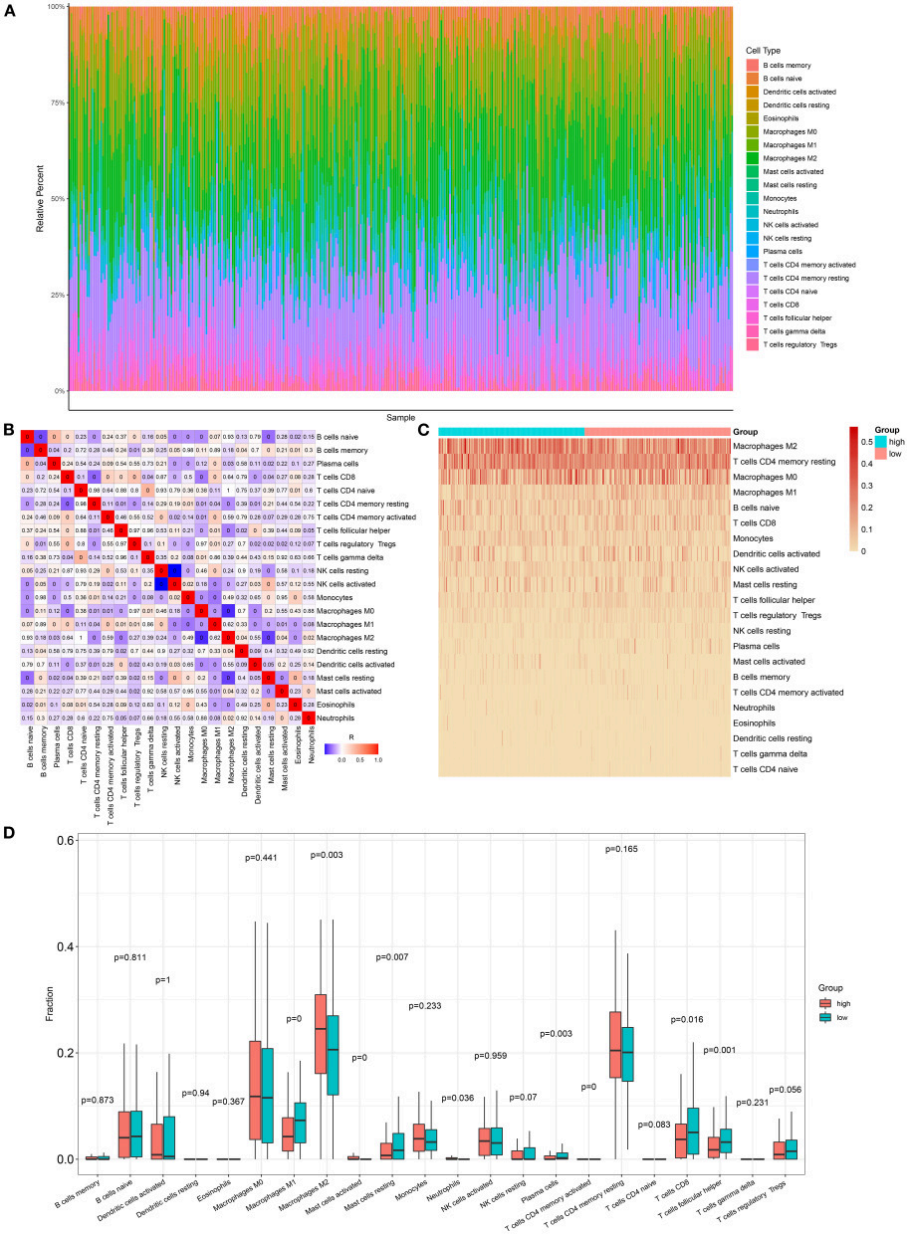
Association between the autophagy-related lncRNA signature and immune cell infiltration in ovarian cancer. **(A)** The proportions of different immune cell components in ovarian cancer tissues. **(B)** Heatmap for the correlations between different immune cells. The darker the color, the greater the |correlation coefficient|. Red: positive correlation and blue: negative correlation. **(C)** Heatmap for the infiltration levels of immune cells in the high- and low-risk samples with ovarian cancer. **(D)** Box plots for the differences in infiltration levels of immune cells between the high- and low-risk samples with ovarian cancer.

Here, we adopted the CIBERSORT algorithm to infer the immune cell infiltration in samples with ovarian cancer. Figure 5A visualized the proportions of 22 kinds of immune cell components in ovarian cancer tissues. Also, we analyzed the correlations between distinct immune cells. In Figure 5B, there were strong correlations between B cells *naïve* and macrophages M2 (*r* = 0.93), between B cells memory and monocytes (*r* = 0.98), between T cells CD4 *naïve* and T cells CD4 memory resting (*r* = 0.98), between T cells follicular helper and Tregs (*r* = 0.97), between T cells follicular helper and T cells gamma delta (*r* = 0.96), between Tregs and macrophages M0 (*r* = 0.97), between T cells gamma delta and mast cells activated (*r* = 0.92), and between monocytes and mast cells activated (*r* = 0.95) in ovarian cancer tissues. Also, heatmap visualized the differences in the immune cell infiltrations between high- and low-risk samples of the ovarian cancer (Figure 5C). Compared with the low-risk group, there were lowered infiltration levels of macrophages M1 (*p* < 0.0001), mast cells resting (*p* = 0.007), plasma cells (*p* = 0.003), T-cell CD8 (*p* = 0.016), and T-cell follicular helper (*p* = 0.001) and also higher infiltration levels of macrophages M2 (*p* = 0.003), mast cells activated (*p* < 0.0001), and neutrophils (*p* = 0.036) in the high-risk group (Figure 5D).

### The Autophagy-Related lncRNA Signature Is Associated With Immune Checkpoints in Ovarian Cancer

**Figure 6:**
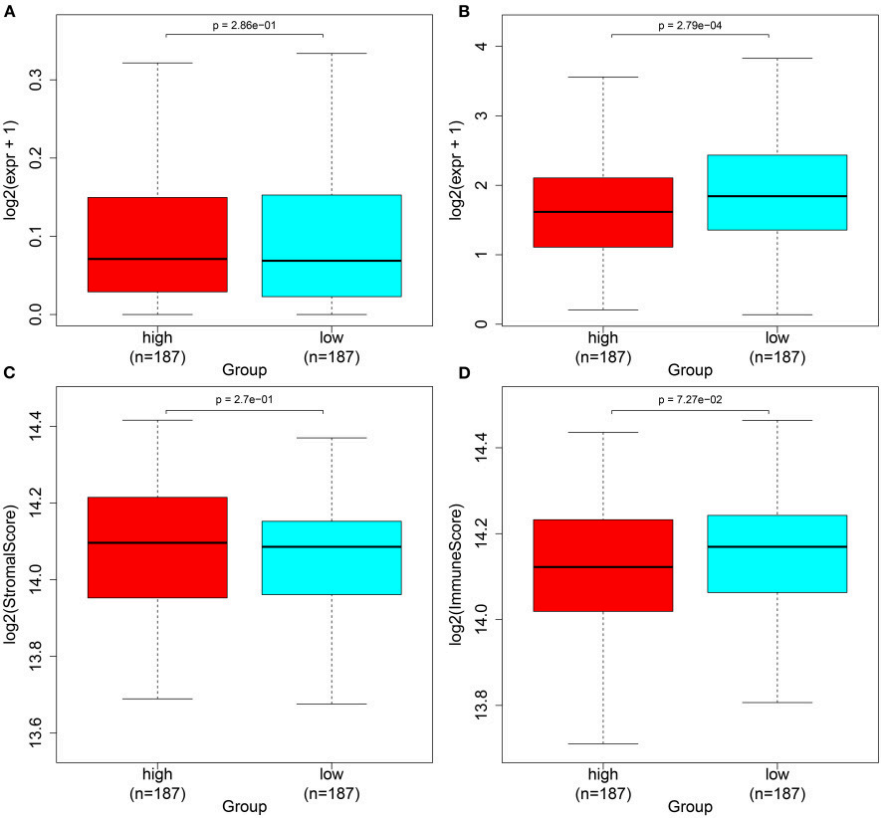
Associations between the autophagy-related lncRNA signature and immune checkpoints in ovarian cancer. **(A,B)** Box plots for the expression of immune checkpoints **(A)** LSECtin and **(B)** PD-L1 in the high- and low-risk groups. **(C,D)** Box plots for the **(C)** immune and **(D)** stromal scores in the high- and low-risk groups.

Herein, we observed whether the autophagy-related lncRNA signature was associated with the expression of immune checkpoints in ovarian cancer tissues. Higher LSECtin expression was found in the high-risk group compared with the low-risk group (Figure 6A). Furthermore, there was distinctly decreased PD-L1 expression in the low-risk group compared with the high-risk group (*p* = 2.79e-04; Figure 6B). We also assessed the correlations between the risk scores and immune or stromal scores. As a result, higher immune scores and lower stromal scores were detected in the high-risk samples compared with the low-risk samples, which were not statistically significant (Figures 6C,D).

### Prediction of the Sensitivity to Chemotherapy Drugs Based on the Autophagy-Related lncRNA Signature

**Figure 7:**
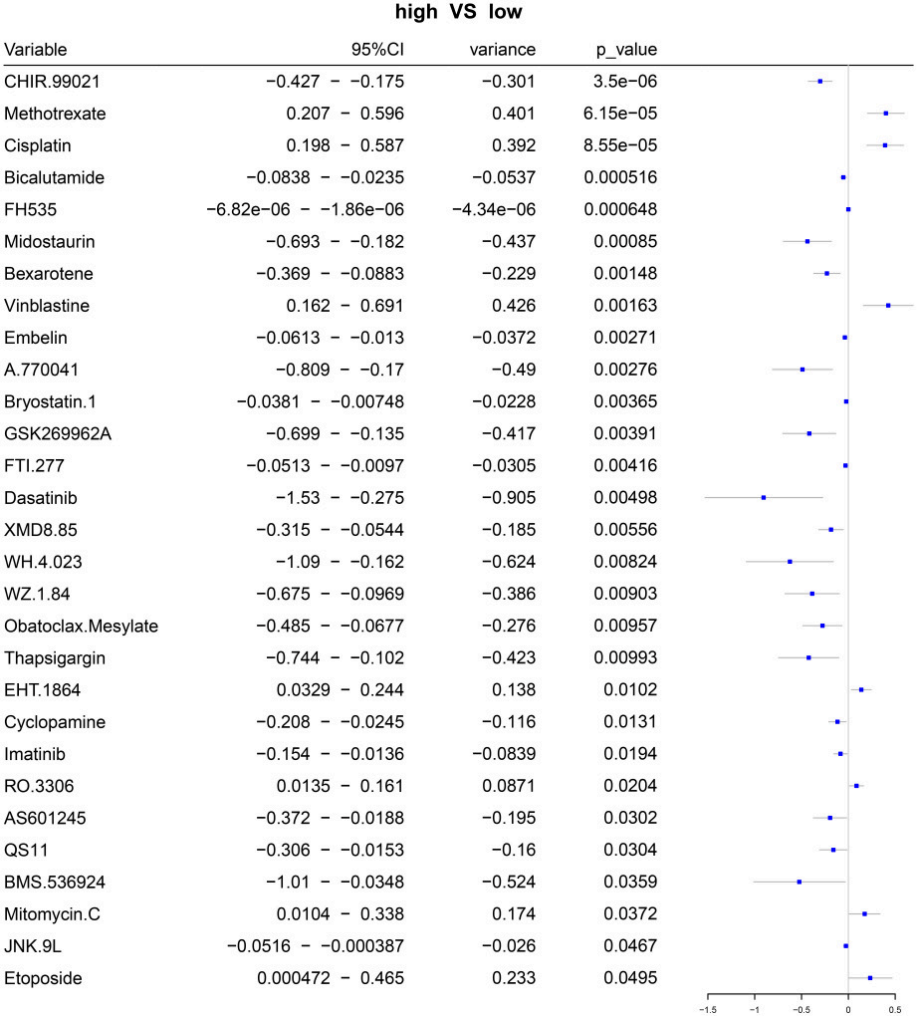
Forest plots for the differences in IC50 values of chemotherapy drugs between the high- and low-risk samples with ovarian cancer.

Based on the gene expression profiles, we predicted the responses to 94 chemotherapy drugs in each patient with ovarian cancer. Among them, there were significant differences in responses to 29 chemotherapy drugs with *p* < 0.05 between high- and low-risk patients with ovarian cancer, including CHIR.99021, methotrexate, cisplatin, bicalutamide, FH535, midostaurin, bexarotene, vinblastine, embelin, A.770041, bryostatin.1, GSK269962A, FTI.277, dasatinib, XMD8.85, WH.4.023, WZ.1.84, Obatoclax.Mesylate, thapsigargin, EHT.1864, cyclopamine, imatinib, RO.3306, AS601245, QS11, BMS.536924, mitomycin.C, JNK.9L, and etoposide (Figure 7; Supplementary Table 4).

### Somatic Mutation Landscapes in Ovarian Cancer

**Figure 8:**
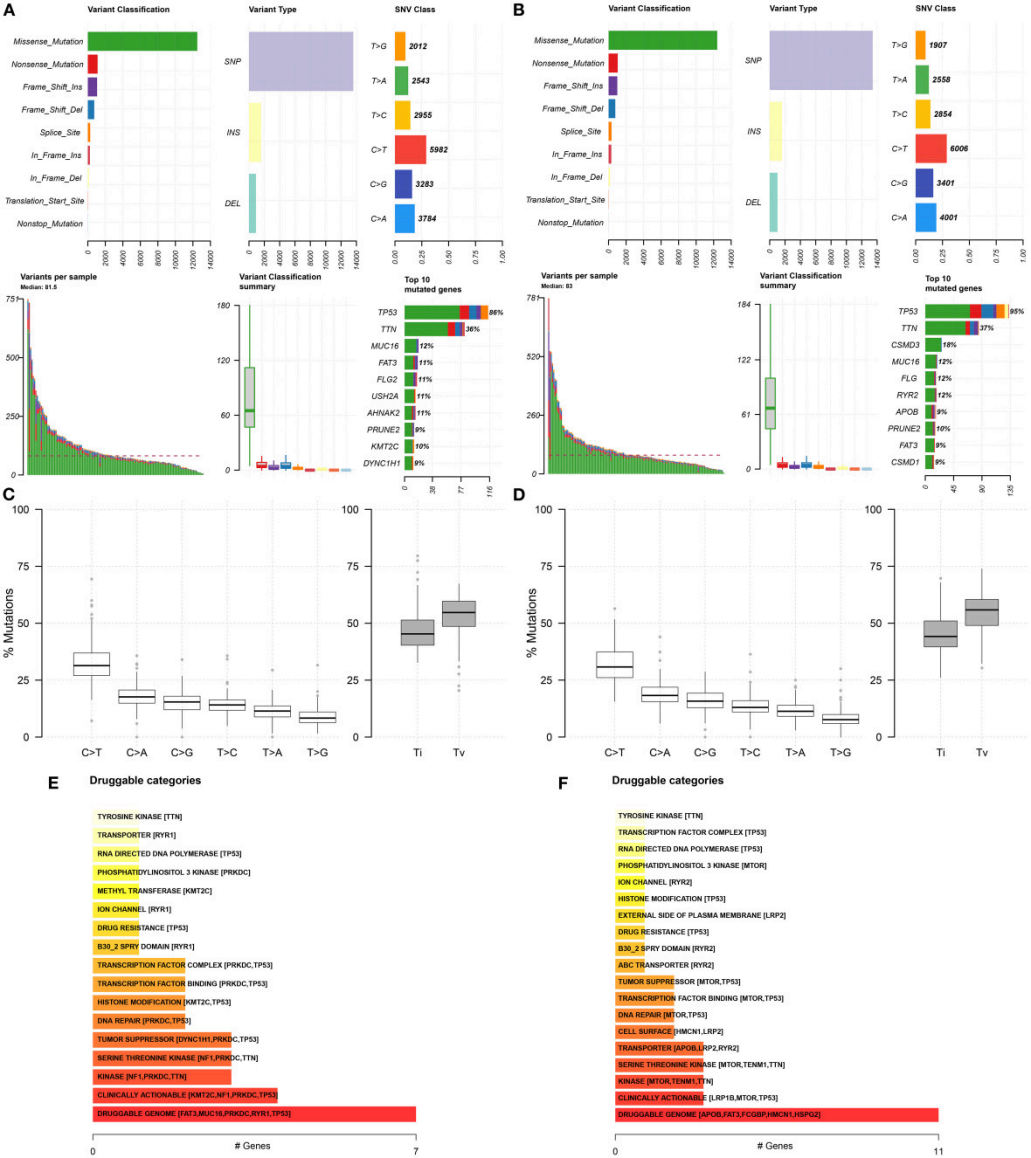
Somatic mutation landscapes in ovarian cancer. **(A,B)** The summary of mutation types and mutated genes in the high- and low-risk samples with ovarian cancer. **(C,D)** The overall distribution of the six different Single-nucleotide variants and the proportion of transitions in the high- and low-risk samples with ovarian cancer. **(E,F)** Druggable categories based on the mutated genes in the high- and low-risk samples.

**Figure 9:**
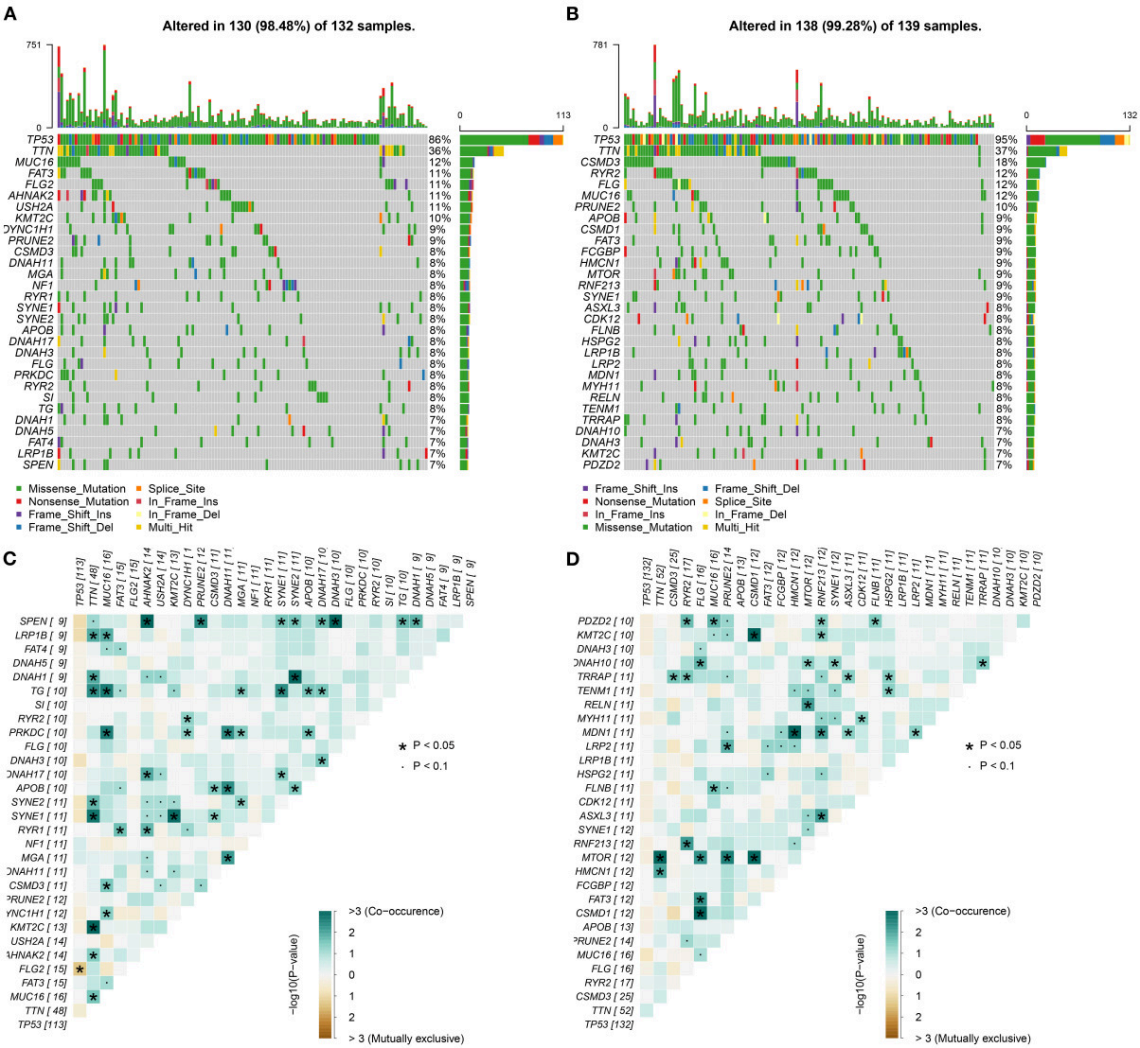
Mutated genes and their interactions in ovarian cancer. **(A,B)** Oncoplots for the top 30 mutated genes in the high- and low-risk samples with ovarian cancer. **(C,D)** The interactions between the mutated genes. The darker the color, the stronger the co-occurrence.

We further analyzed the somatic mutation landscapes in specimens of ovarian cancer. Both in the high- (Figure 8A) and low-risk (Figure 8B) samples, missense mutation was the most common mutation type. TP53 was the top-ranked mutated gene, followed by TTN. C > T mutation had the highest proportion in the two groups (Figures 8C,D). Furthermore, potential druggable categories targeted mutated genes were predicted, such as the druggable genome, clinically actionable, and kinase in the high- (Figure 8E) and low-risk (Figure 8F) samples. Approximately 98.48% of samples occurred genetic mutations in the high-risk samples (Figure 9A) and 99.28% of the occurred mutations in the low-risk samples (Figure 9B). Many genes co-occur in cancer or show strong exclusivity in their mutation patterns. Here, we visualized the close interactions between the mutated genes in the high- and low-risk samples (Figures 9C,D).

### Significant Signaling Pathways Underlying the Autophagy-Related lncRNA Signature

**Figure 10:**
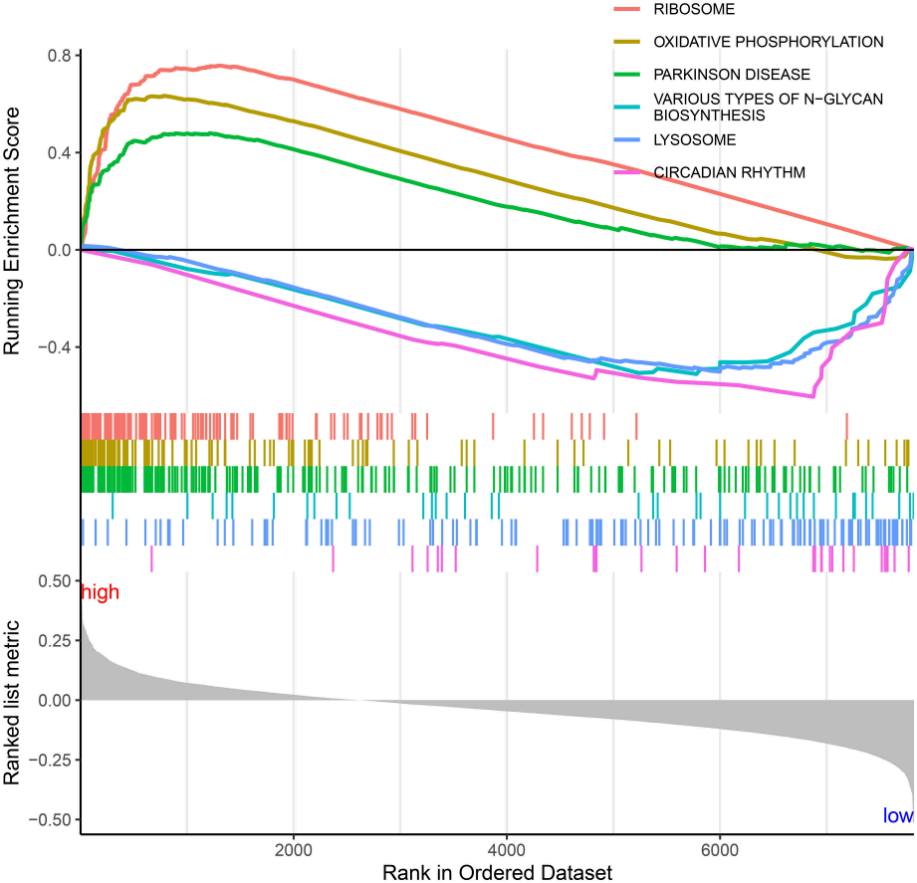
Significant signaling pathways underlying the autophagy-related lncRNA signature by Gene set enrichment analysis.

To explore the significant signaling pathways underlying the autophagy-related lncRNA signature, this study carried out GSEA by comparing high- and low-risk groups. As shown in Figure 10, we observed that ribosome (NES = 1.90 and nominal *p* = 0.019), oxidative phosphorylation (NES = 1.76 and nominal *p* = 0.037), and Parkinson's disease (NES = 1.74 and nominal *p* = 0.035) were markedly activated in the high-risk group. Meanwhile, various types of N-glycan biosynthesis (NES = −1.84 and nominal *p* = 0.002), lysosome (NES = −1.87 and nominal *p* = 0.002), and circadian rhythm (NES = −2.08 and nominal *p* < 0.0001) were enriched significantly in the low-risk group.

## Discussion

As per a previous study, 17 autophagy-related lncRNAs have been identified as prognostic predictors of ovarian cancer (26). Nevertheless, a single autophagy-related lncRNA often has limited predictive power. Furthermore, the established clinical prognostic biomarkers have limited accuracy and specificity. It has been confirmed that gene models exhibit higher predictive power than a single gene (5). This study employed the LASSO regression method to establish an autophagy-related lncRNA signature containing AC084018.1, AC092171.2, AP000695.1, GAS6-AS1, LINC00174, LINC00893, LINC00996, MEIS1-AS3, MIR22HG, NEAT1, TEX26-AS1, U73169.1, UBE2Q1-AS1, and USP30-AS1 for ovarian cancer based on abnormally expressed autophagy-related lncRNA profiles. After verification, this signature robustly and independently predicted the survival outcomes of the patients with ovarian cancer. Among the 14 lncRNAs in this signature, GAS6-AS1 exerts a carcinogenic effect in the breast cancer (27) and hepatocellular carcinoma (28). LINC00174 promotes glioma progression *via* miR-152-3p/SLC2A1 axis (29). LINC00893 suppresses papillary thyroid cancer by inactivating the AKT pathway and stabilizing PTEN (30). USP30-AS1 expression is related to autophagy in the bladder urothelial carcinoma (31). More experiments require to verify the regulatory roles of these lncRNAs on autophagy.

As immunogenic cancers, the spontaneous anticancer immune responses may increase survival duration, and the immune escape may cut down survival duration (32). In the recent years, several immune-based strategies such as immune checkpoint inhibition, vaccination, and antigen-specific active immunotherapy have been developed in ovarian cancer (33). The immune-suppressive networks in the tumor microenvironment have been considered for immunotherapy implementation (34). The immune-related markers may be utilized for predicting the responses to immunotherapy (35). Hence, the interactions between molecules and tumor microenvironment require in-depth exploration. Our study demonstrated that the autophagy-related lncRNA signature was in relation to infiltrations of macrophages M1, mast cells resting, plasma cells, T-cell CD8, T-cell follicular helper, macrophages M2, mast cells activated, and neutrophils, indicating the crosstalk between these autophagy-related lncRNAs and immune cells. Consistently, Deng et al. identified a novel autophagy-related lncRNA model that was distinctly related to the infiltrations of immune cells in pancreatic cancer (36). Ovarian cancer represents a low immune-reactive malignancy with restricted immune cell infiltrations and extensive immunosuppressive T cell infiltrations (37). Tumors positive for PD-L1 usually exhibit a higher response to immune checkpoint inhibition therapy, and highly expressed PD-L1 is a predictor of undesirable clinical outcomes (37). Here, our data showed that the low-risk ovarian cancer patients had higher PD-L1 expression in comparison to those with high risk.

Chemotherapy (platinum and taxanes) plays a fundamental role in adjuvant therapy against ovarian cancer (38). Despite the initial response to this therapy, most of the patients diagnosed with ovarian cancer developed chemotherapy resistance (39). This resistance may be driven by a range of mechanisms. Hence, it is of importance to develop individual markers for the prediction of the sensitivity to chemotherapy. Our data showed that the risk scores were in relation to 29 chemotherapy drugs such as cisplatin, indicating that the autophagy-related lncRNAs were involved in chemotherapy sensitivity, as a previous study (11).

## Conclusions

Collectively, this study provided a knowledge base of novel autophagy-related lncRNAs in ovarian cancer, improving the understanding of the functions of lncRNA on the regulation of autophagy in ovarian cancer. We established an autophagy-related lncRNA signature as a robust prognostic marker for the prediction of survival outcomes, immunotherapy response, and chemotherapy sensitivity. Our findings may assist to precisely guide therapeutic strategies for individual patients with ovarian cancer in clinical practice.

## Data Availability Statement

The original contributions presented in the study are included in the article/Supplementary Material, further inquiries can be directed to the corresponding author/s.

## Author Contributions

JiW conceived and designed the study. YL and JuW conducted most of the experiments and data analysis and wrote the manuscript. FW, CG, and YC participated in collecting data and helped to draft the manuscript. All authors reviewed and approved the manuscript.

## Conflict of Interest

The authors declare that the research was conducted in the absence of any commercial or financial relationships that could be construed as a potential conflict of interest.

## Publisher's Note

All claims expressed in this article are solely those of the authors and do not necessarily represent those of their affiliated organizations, or those of the publisher, the editors and the reviewers. Any product that may be evaluated in this article, or claim that may be made by its manufacturer, is not guaranteed or endorsed by the publisher.

## Supplementary Material

Supplementary Table 1 The list of upregulated lncRNAs in ovarian cancer tissues.

Supplementary Table 2 The list of downregulated lncRNAs in ovarian cancer tissues.

Supplementary Table 3 The correlation analysis results between abnormally expressed lncRNAs and autophagy-related genes.

Supplementary Table 4 The list of chemotherapy drugs.

## Abbreviations

LncRNAs: long non-coding RNAs
TCGA: The Cancer Genome Atlas
GTEx: Genotype-Tissue Expression
FC: fold change
LASSO: least absolute shrinkage and selection operator
OS: overall survival
HR: hazard ratio
ROC: receiver operating characteristic
ESTIMATE: Estimation of STromal and Immune cells in Malignant Tumor tissues using Expression data.
